# Non‐Coding RNAs in Breast Cancer Radioresistance: Mechanisms, Functional Roles and Translational Potentials

**DOI:** 10.1111/cpr.70119

**Published:** 2025-09-14

**Authors:** Xiaohui Zhao, Yuting Qiu, Jie Chen, Danni Wang, Zairui Wang, Shuang Ma, Yimin Liu, Guoying Liu, Zhuofei Bi

**Affiliations:** ^1^ Department of Radiation Oncology, Sun Yat‐sen Memorial Hospital Sun Yat‐sen University Guangzhou China; ^2^ Department of Radiation Oncology, Shenshan Medical Centre Memorial Hospital of Sun Yat‐sen University Shanwei China; ^3^ Guangdong Provincial Key Laboratory of Malignant Tumor Epigenetics and Gene Regulation, Sun Yat‐sen Memorial Hospital Sun Yat‐sen University Guangzhou China

**Keywords:** breast cancer, ncRNAs, radioresistance, radiotherapy, treatment strategies

## Abstract

Breast cancer remains the most prevalent malignancy among women, and radiotherapy plays a pivotal role in reducing local recurrence and improving prognosis. However, the emergence of radioresistance in a subset of patients significantly compromises treatment efficacy, underscoring the need for a deeper understanding of the underlying molecular mechanisms. In recent years, non‐coding RNAs (ncRNAs) have emerged as key regulators of gene expression and have garnered increasing attention for their roles in mediating radioresistance in breast cancer. This review systematically summarises the major molecular mechanisms by which ncRNAs contribute to breast cancer radioresistance, including cell cycle regulation, DNA damage repair, programmed cell death (e.g., apoptosis, autophagy and ferroptosis), oxidative stress response, tumour microenvironment remodelling and maintenance of cancer stem cell properties. On the translational front, RNA‐based therapeutic approaches—including antisense oligonucleotides (ASOs), small interfering RNAs (siRNAs), miRNA mimics and CRISPR/Cas9—offer promising avenues for radiosensitisation, yet face substantial clinical hurdles. These include immune activation, poor delivery specificity, intracellular trafficking barriers and limited stability. Advances in chemical modifications and nanoparticle‐based delivery systems—such as redox‐responsive nanocarriers—have shown potential in enhancing the efficacy and safety of ncRNA‐targeted therapies. Despite encouraging progress, clinical translation remains constrained by a lack of methodological standardisation, insufficient high‐quality clinical data, limited biomarker reliability, suboptimal target selection and unresolved safety concerns. Future efforts should prioritise optimisation of delivery platforms, validation of multi‐ncRNA biomarker panels in large, multicentre cohorts and integration of multi‐omics data to reconstruct comprehensive regulatory networks, ultimately accelerating the clinical deployment of ncRNA‐based radiosensitisation strategies.

## Graphical abstract

Non‐coding RNAs (ncRNAs) regulate breast cancer radioresistance via cell cycle, DNA repair and tumour microenvironment pathways. Targeting ncRNAs (e.g., HOTAIR and miR‐155) with RNA‐based therapies (ASOs and CRISPR) shows promise but faces delivery challenges. Advances in nanocarriers and multi‐omics integration may overcome barriers to clinical translation of ncRNA radiosensitisation strategies.

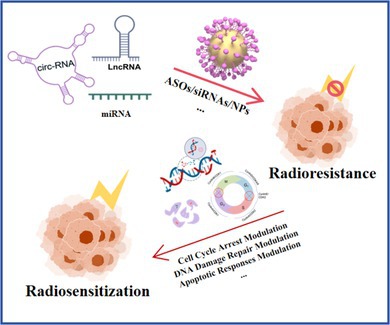

**Abbreviations**
3′‐UTRs: 3′ untranslated regions
ABCC1: ATP‐binding cassette sub‐family C member 1
AGO2: Argonaute 2
AIFM2: apoptosis‐inducing factor mitochondria‐associated 2
AMPK: AMP‐activated protein kinase
ASOs: antisense oligonucleotides
ATM: ataxia‐telangiectasia mutated
ATR: ATM and Rad3‐related
BRCA1: breast cancer type 1 susceptibility protein
BRCA2: breast cancer type 2 susceptibility protein
CAFs: cancer‐associated fibroblasts
CAT: catalase
CCAT1: colon cancer‐associated transcript 1
CDK1: cyclin‐dependent kinase 1
CDK2: cyclin‐dependent kinase 2
ceRNA: competing endogenous RNA
CHK1: Checkpoint Kinase 1
circRNA: circular RNA
CPPs: cell‐penetrating peptides
cRBPs: circular RNA‐binding proteins
CSCs: cancer stem cells
DDR: DNA damage response
D‐loop: displacement loop
DNA‐PKcs: DNA‐dependent protein kinase
DSBs: double‐strand breaks
ECM: extracellular matrix
EGFR: epidermal growth factor receptor
EMT: epithelial–mesenchymal transition
ERK: extracellular signal‐regulated kinase
FasL: Fas Ligand
FGD5‐AS1: FGD5 antisense transcript 1
FOXO3: Forkhead box O3
G3BP1: Ras GTPase‐activating protein‐binding protein 1
GPx: glutathione peroxidase
HCG18: HLA complex group 18
HIF‐1: hypoxia‐inducible factor‐1
HMGB1: High Mobility Group Box 1
HR: homologous recombination
HRR: homologous recombination repair
IGF2BP1: insulin‐like growth factor 2 mRNA‐binding protein 1
IR: ionising radiation
LC3‐I: microtubule‐associated protein 1A/1B‐light chain 3‐I
LLPS: liquid–liquid phase separation
lncRNA NEAT: lncRNA 1nuclear paraspeckle assembly transcript 1
lncRNA RUNX1‐IT1: lncRNA RUNX1 intronic transcript 1
lncRNA UCA1: lncRNA urothelial cancer associated 1
lncRNAs: long non‐coding RNAs
m6A: N6‐methyladenosine
MACC1: metastasis‐associated in colon cancer 1
MDM2: mouse double minute 2 homologue
MET: mesenchymal–epithelial transition
miRNAs: microRNAs
mTOR: mammalian target of rapamycin
ncRNAs: non‐coding RNAs
NHEJ: non‐homologous end joining
NPs: Nanoparticles
PALB2: partner and localizer of BRCA2
PCAT6: Prostate cancer‐associated transcript 6
p‐EGFR: phosphorylated epidermal growth factor receptor
PFN2: Profilin 2
PI3K/AKT: phosphoinositide 3‐kinase/protein kinase B
PIP2: phosphatidylinositol‐4,5‐bisphosphate
PIP3: phosphatidylinositol‐3,4,5‐trisphosphate
PMN: pre‐metastatic niche
PRC2: Polycomb Repressive Complex 2
PTEN: Phosphatase and Tensin Homologue deleted on chromosome 10
PUFAs: polyunsaturated fatty acids
RISC: RNA‐Induced Silencing Complex
ROC: receiver operating characteristic
ROS: reactive oxygen specie
RPA: replication protein A
RTKs: receptor tyrosine kinases
SGPP1: sphingosine‐1‐phosphate phosphatase 1
siRNAs: small interfering RNAs
SIRT1: Sirtuin 1
SLC7A11: solute carrier family 7 member 11
SNARE: soluble N‐ethylmaleimide‐sensitive factor attachment protein receptor
SNHG5: small nucleolar RNA host gene 5
SOD: superoxide dismutase
SPP1: Secreted Phosphoprotein 1
SSBs: single‐strand breaks
ssDNA: single‐stranded DNA
TAMs: tumour‐associated macrophages
TBK1: TANK‐binding kinase 1
TLR4: toll‐like receptor 4
TME: tumour microenvironment
TNBC: triple‐negative breast cancer
TPBG: trophoblast glycoprotein
TPD52: tumour protein D52
TRAIL: TNF‐related apoptosis‐inducing ligand
Tregs: regulatory T cells
UBE2O: ubiquitin‐conjugating enzyme E2O
UBE3C: ubiquitin protein ligase 3C
ULK1: Unc‐51‐like autophagy activating kinase 1
VPS34: Vacuolar Protein Sorting 34
ZEB1: Zinc finger E‐box binding homeobox 1
ZEB2: zinc finger E‐box‐binding homeobox 2
ZNF281: zinc finger protein 281

## 1 Introduction

Breast cancer remains the most frequently diagnosed malignancy among women worldwide [1]. In 2024, it is projected to account for 32% of all newly diagnosed invasive cancers in women in the United States, according to the American Cancer Society [2]. In China, breast cancer represented 15.6% of new cancer cases among women in 2022, with an estimated 357,200 cases [3]. As one of the most prevalent cancers globally, advances in standardised multimodal therapies have substantially improved patient outcomes [4]. Radiotherapy plays an essential role in breast cancer management, particularly as adjuvant treatment following breast‐conserving surgery, where it significantly reduces the risk of local recurrence [5]. Despite its well‐established clinical benefit, a subset of patients still develops local relapse or disease progression after radiotherapy, highlighting the presence of intrinsic or acquired radioresistance. This therapeutic resistance undermines treatment efficacy and remains a major clinical hurdle to improving long‐term survival [6].

The mechanisms underlying radioresistance are multifactorial and complex, encompassing genetic and epigenetic alterations, dynamic interactions within the tumour microenvironment (TME) and intratumoral heterogeneity [7]. In recent years, non‐coding RNAs (ncRNAs)—a diverse class of transcripts lacking protein‐coding potential but endowed with pivotal regulatory capacity—have emerged as key modulators of cancer biology [8, 9]. Notably, ncRNAs constitute over 90% of the human transcriptome and are increasingly recognised for their roles in tumour initiation, progression, metastasis, apoptosis, stemness, angiogenesis and resistance to therapy in multiple malignancies, including breast cancer [10, 11].

In this review, we provide a comprehensive overview of the current understanding of ncRNAs in breast cancer radioresistance, with a focus on their regulatory mechanisms and the signalling networks they engage. We discuss the emerging potential of ncRNAs as diagnostic biomarkers and therapeutic targets and highlight recent advances that offer mechanistic insights and translational implications. By integrating findings from basic, preclinical and clinical research, this review aims to illuminate novel strategies to enhance radiosensitivity and enable precision radiotherapy in breast cancer.

### 1.1 Mechanisms of Tumour Cell Killing by Ionising Radiation (IR) and Pathways of Radioresistance

**FIGURE 1:**
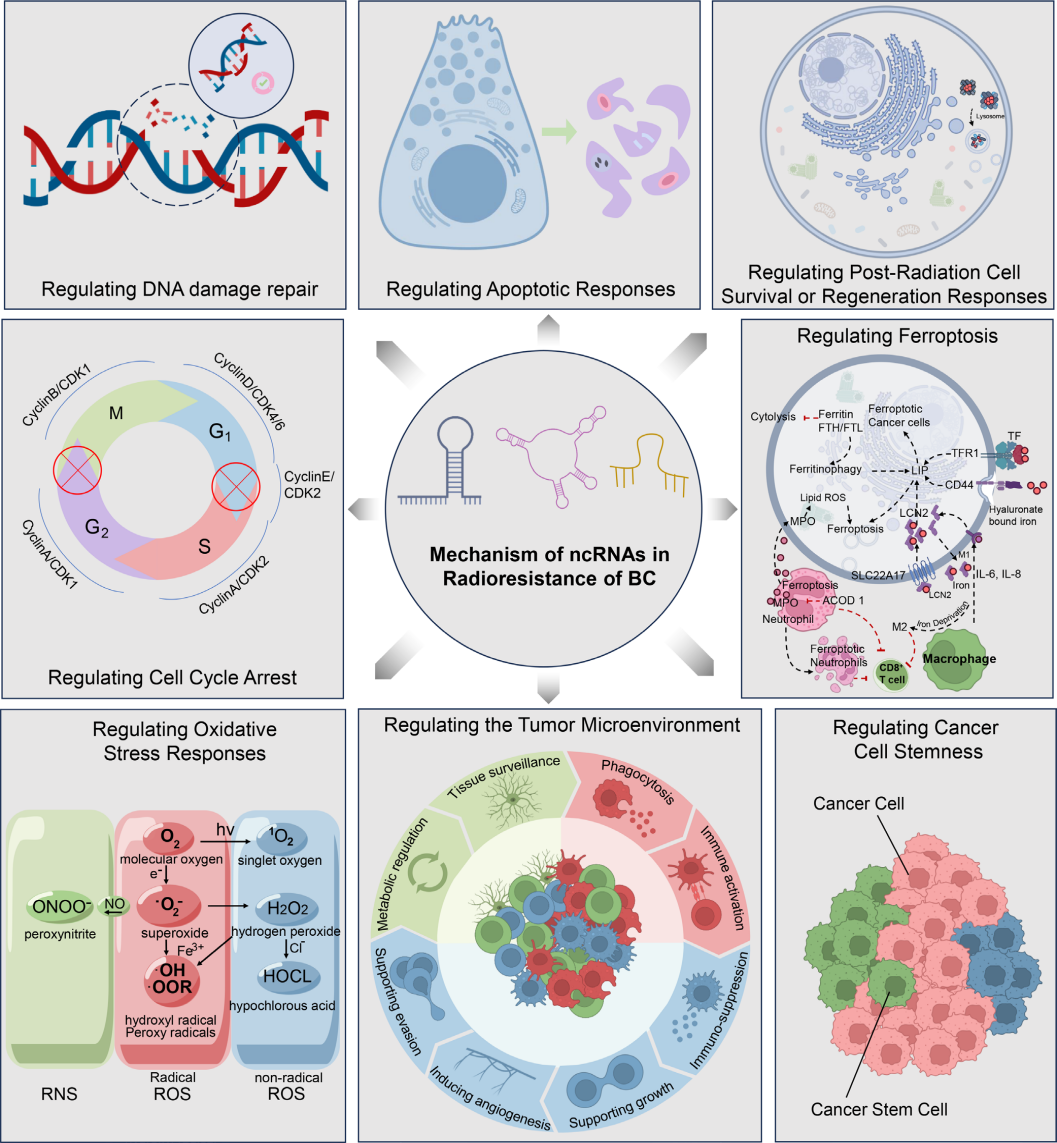
Mechanisms of ncRNAs in radioresistance of breast cancer. The potential mechanisms by which ncRNAs contribute to radiotherapy resistance in breast cancer include the regulation of cell cycle arrest, DNA damage repair, apoptotic responses, post‐radiation cell survival or regeneration responses, oxidative stress responses, the tumour microenvironment, cancer cell stemness and ferroptosis.

As illustrated in Figure 1, ncRNAs regulate breast cancer radioresistance through multiple key mechanisms.

#### 1.1.1 Direct and Indirect Mechanisms of IR‐Induced Cell Death

IR is a cornerstone of modern cancer therapy, exerting cytotoxic effects through both direct and indirect mechanisms [12]. The direct effect involves the interaction of high‐energy radiation with cellular DNA, resulting in the generation of DNA double‐strand breaks (DSBs)—one of the most lethal forms of DNA damage. These lesions disrupt genomic integrity, trigger cell cycle arrest and, if irreparable, initiate apoptosis or permanent proliferative arrest, leading to tumour cell death [13].

In contrast, the indirect effects of IR primarily involve the radiolysis of water molecules within cells, generating reactive oxygen species (ROS) such as hydroxyl radicals (·OH), superoxide (O_2_^−^) and hydrogen peroxide (H_2_O_2_) [14]. These ROS inflict oxidative damage on critical biomolecules, including DNA, lipids and proteins, thereby impairing essential cellular functions and promoting cell death [15]. Importantly, while ROS contribute to radiation‐induced cytotoxicity, they also activate pro‐survival and stress response pathways, which may paradoxically promote tumour cell survival and adaptive radioresistance [16].

The spectrum of IR‐induced DNA damage includes base modifications, single‐strand breaks (SSBs) and DSBs. Base damage is typically mediated by oxidative stress, whereas SSBs and DSBs are more directly linked to the energy deposited by radiation [17]. Among these, DSBs are the most deleterious, as their misrepair or persistence can lead to chromosomal aberrations, mitotic catastrophe, or programmed cell death [18]. The ability of tumour cells to repair DNA damage is a critical determinant of radiosensitivity. Due to tumour heterogeneity, variations in DNA damage response (DDR) and repair efficiency across different subclones may result in differential radiation responses, contributing to treatment failure and disease recurrence [13].

Taken together, the cytotoxicity of IR arises from a complex interplay between direct DNA damage and ROS‐mediated oxidative stress. A deeper understanding of these mechanisms not only enables the refinement of radiotherapy regimens but also informs the development of novel radiosensitisation strategies. For instance, targeting redox homeostasis or inhibiting specific DNA repair pathways could potentiate radiation efficacy and overcome intrinsic or acquired radioresistance.

#### 1.1.2 Tumour Cells Confer Radioresistance via Cell Cycle Arrest Modulation

Regulation of the cell cycle plays a pivotal role in tumour cell resistance to radiotherapy [19]. The eukaryotic cell cycle is divided into four distinct phases—G1, S, G2 and M—each monitored by specific checkpoints that ensure genomic integrity and proper cellular growth [20]. Among them, the G1/S and G2/M checkpoints are especially critical in mediating the cellular response to DNA damage induced by IR [19].

Upon irradiation‐induced DNA damage, cells activate the ataxia‐telangiectasia mutated (ATM) and ATM and Rad3‐related (ATR) kinases, initiating a cascade of DDR signalling. This leads to the activation of downstream checkpoint kinases—checkpoint kinase 2 (CHK2) and checkpoint kinase 1 (CHK1), respectively. These checkpoint kinases inhibit the activity of the cell division cycle 25A (CDC25A) and CDC25C phosphatases, thereby suppressing the activity of cyclin‐dependent kinase 2 (CDK2) and cyclin‐dependent kinase 1 (CDK1), which are essential for cell cycle progression. The resulting cell cycle arrest at the G1/S or G2/M checkpoint provides a temporal window for DNA repair and allows tumour cells to evade radiation‐induced cell death [21, 22].

**FIGURE 2:**
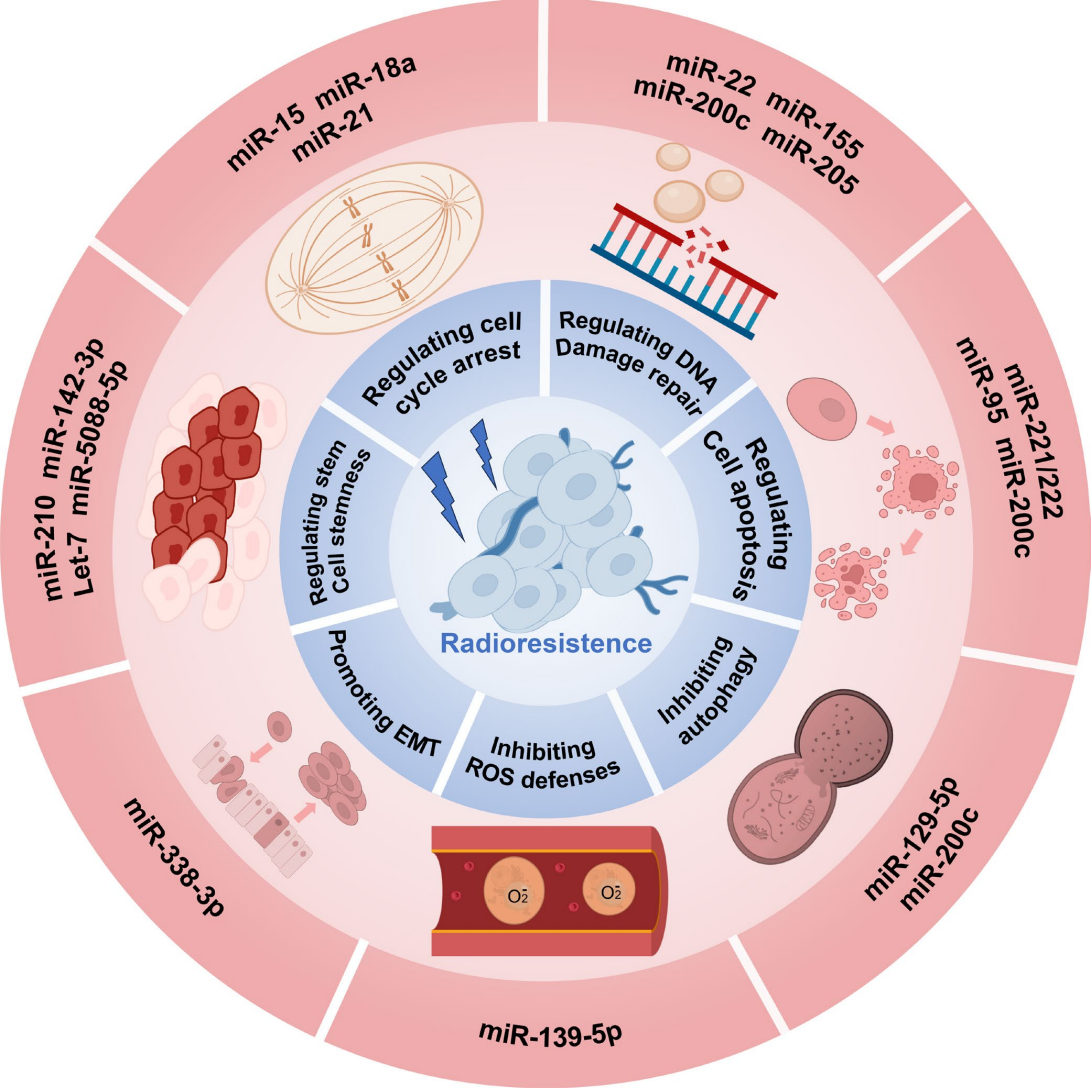
The primary mechanisms by which miRNAs influence radioresistance in breast cancer cells. (1) miR‐15, miR‐18a and miR‐21 directly or indirectly regulate cell cycle arrest by modulating enzymes or molecules associated with cell cycle checkpoints, thereby affecting the sensitivity of breast cancer cells to radiotherapy. (2) miR‐22, miR‐155, miR‐200c and miR‐205 influence the response of breast cancer cells to radiotherapy by regulating DNA repair factors and chromatin remodelling complexes associated with DNA repair pathways. (3) miR‐221/222, miR‐95 and miR‐200c primarily influence the sensitivity of breast cancer cells to radiotherapy by regulating apoptotic or anti‐apoptotic pathways. (4) Autophagy has evolved into a crucial mechanism for cancer cell survival, contributing to resistance against various treatments, including radiotherapy. miR‐129‐5p and miR‐200c decrease their radiation resistance by inhibiting autophagy. (5) Tumour cells produce high levels of antioxidants to eliminate excess reactive oxygen species (ROS) and maintain intracellular redox balance, which supports cell survival and ultimately contributes to radiation resistance. miR‐139‐5p enhances the sensitivity of breast cancer to radiotherapy by inhibiting a multi‐gene network involved in ROS defence. (6) Hypoxia promotes radioresistance in tumour cells through mechanisms such as facilitating epithelial–mesenchymal transition (EMT). It enhances radiation resistance by inhibiting the transcription of miR‐338‐3p, which is a promoter of EMT. (7) Cancer stem cells contribute to radiotherapy resistance, with miR‐210, miR‐142‐3p, Let‐7 and miR‐5088‐5p influencing the radiosensitivity of breast cancer cells by regulating stemness.

Accumulating evidence suggests that multiple ncRNAs modulate the extent of cell cycle arrest by targeting critical regulatory proteins, thereby influencing radiosensitivity. For instance, Mei et al. reported that members of the miR‐15 family suppress the expression of CHK1 and Wee1 by binding to their 3′ untranslated regions (3′‐UTRs). Wee1 is a serine/threonine kinase that phosphorylates CDK1 at Tyr15 to inhibit mitotic entry, thus reinforcing the G2/M checkpoint. Downregulation of CHK1 and Wee1 by miR‐15 reduces G2/M arrest, thereby enhancing the radiosensitivity of breast cancer cells [23] (Figure 2, Table 1).

**TABLE 1:** The roles of specific miRNAs in regulating radiosensitivity and radioresistance of BC are examined. Each miRNA, along with its target molecule and its function in the radiation response, is detailed.

miRNAs	Target	Notes	References
miR‐15	Chk1, Weel	The miR‐15 family suppresses G2/M cell cycle arrest and enhances radiosensitivity of BC cells by inhibiting Chk1 and Weel	[23]
miR‐18a	ATM	Overexpression of miR‐18 abrogates IR‐induced cell cycle arrest and enhances the radiosensitivity of BC cells by downregulating ATM expression	[24]
*miR‐21*	G2/M checkpoint	miR‐21 expression in BC cells contributes to radiation resistance via G2/M checkpoint arrest	[25]
miR‐200c	LINC02582/USP7/CHK1 signalling axis	miR‐200c increases radiosensitivity via LINC02582/USP7/CHK1 signalling axis	[26]
	EGFR	The overexpression of miR‐200c increases the radiosensitivity of cancer cells via EGFR‐associated pro‐survival signalling	[27]
	TBK1	miR‐200c improves the sensitivity of breast cancer cells to radiotherapy by directly targeting TBK1	[28]
	UBQLN1	miR‐200c targets Ubiquilin 1 (UBQLN1), thereby enhancing radiosensitivity in breast cancer cells.	[29]
miR‐22	Sirt1	miR‐22 improves radiosensitivity of BC cells by targeting Sirt1	[30]
miR‐155	RAD51	Overexpression of miR‐155 reduces HR repair efficiency and enhances sensitivity to IR by targeting RAD51	[31]
miR‐205	ZEB1, Ubc13	miR‐205 increases radiosensitivity by directly targeting ZEB1 and Ubc13, thereby inhibiting DNA damage repair	[32]
miR‐944	PI3K/AKT/SPP1 pathway	miR‐944 suppresses BC cell proliferation and induces apoptosis by downregulating SPP1 through inhibiting the PI3K/Akt pathway	[33]
let‐7	cyclin D1/Akt1/Wnt1 pathway	let‐7 enhances radiosensitivity by inhibiting the cyclin D1/Akt1/Wnt1 pathway	[34]
*miR‐95*	SGPP1	The overexpression of miR‐95 promotes radiation resistance in breast cancer by targeting SGPP1	[35]
*miRNA‐221/222*	PTEN/AKT pathway	miRNA‐221/222 inhibits IR‐induced apoptosis and the radiation sensitivity by regulating the PTEN/AKT pathway	[36]
*miR‐129‐5p*	HMGB1	miR‐129‐5p sensitised breast cancer cells to irradiation by targeting HMGB1	[37]
miR‐139‐5p	DNA repair and ROS defence genes	miR‐139‐5p increases radiotherapy sensitivity by inhibiting key gene networks involved in DNA repair and ROS defence	[38]
miR‐142‐3p	β‐catenin	miR‐142‐3p is downregulated in BC cells, reducing CSC characteristics and decreasing radioresistance by inhibiting β‐catenin expression	[39]

*Note*: Italics indicate miRNAs that enhance radioresistance, while underlined miRNAs are those that promote radiosensitivity.

In addition, several other miRNAs also participate in modulating DDR and cell cycle checkpoints. For example, miR‐18a directly targets the 3′‐UTR of ATM, attenuating ATM‐dependent checkpoint activation and homologous recombination repair (HRR), ultimately sensitising breast cancer cells to IR [24] (Figure 2, Table 1). miR‐21 is a commonly upregulated miRNA in breast cancer, with levels further increasing after radiotherapy. Anastasov et al. demonstrated that suppression of miR‐21 alleviates IR‐induced G2/M arrest, partially reversing the radioresistant phenotype [25] (Figure 2, Table 1). Additionally, miR‐200c is considered a key factor in regulating breast cancer sensitivity to radiotherapy, with some of its mechanisms involving cell cycle control. Recent studies have found that miR‐200c directly binds to the long non‐coding RNA (lncRNA) LINC02582 through complementary sequences. This process is mediated by Argonaute 2 (AGO2), a core component of the RNA‐Induced Silencing Complex (RISC), leading to the degradation of LINC02582. LINC02582 interacts with the deubiquitinating enzyme USP7 to remove ubiquitination modifications from Checkpoint Kinase 1 (CHK1), thereby extending its protein stability. Thus, miR‐200c can enhance breast cancer cell sensitivity to radiotherapy by regulating the LINC02582–USP7–CHK1 pathway, weakening G2/M checkpoint activity [26] (Figure 2, Table 1).

In addition to miRNAs, circular RNAs (circRNAs) also play a significant role in regulating the cell cycle and radiotherapy sensitivity. Research indicates that circNCOR1 acts as a competing endogenous RNA (ceRNA) by sequestering miR‐638, thereby relieving its inhibition on the downstream target gene CDK2 and resulting in CDK2 upregulation. CDK2 is a key regulatory factor in the G1/S phase transition; its increased expression promotes cell cycle progression, inhibits radiotherapy‐induced apoptosis and reduces breast cancer cell sensitivity to IR [40] (Table 3).

In summary, tumour cells can enhance cell cycle arrest by activating the G1/S or G2/M checkpoints, gaining a time advantage in the DNA damage repair process, which contributes to their resistance to radiotherapy. ncRNAs, such as miRNAs, play an irreplaceable role as key regulatory molecules in the cell cycle and DDR networks, influencing the regulation of radiotherapy responses. In‐depth studies of these ncRNAs not only help uncover the molecular mechanisms underlying radiotherapy resistance in breast cancer but also provide theoretical foundations and potential targets for developing new sensitisation strategies for radiotherapy.

#### 1.1.3 Tumour Cells Confer Radioresistance via Modulation of DNA Damage Repair

DNA damage repair plays a pivotal role in mediating radioresistance in breast cancer. Among radiation‐induced lesions, DSBs represent the most lethal form of damage. To counteract these cytotoxic effects, tumour cells activate distinct DNA repair pathways, thereby diminishing the efficacy of radiotherapy [44]. The two principal mechanisms for DSB repair are homologous recombination (HR) and non‐homologous end joining (NHEJ). HR is a high‐fidelity repair pathway predominantly active during the S and G2 phases of the cell cycle [45, 46], while NHEJ operates throughout all cell cycle stages and is characterised by its rapid but error‐prone nature [47, 48].

Emerging evidence suggests that breast cancer cells can activate both HR and NHEJ in response to radiation‐induced DNA damage, thus enhancing their intrinsic radioresistance. The HR pathway is initiated when ATM kinase recognises DSBs and triggers downstream signalling cascades, including CHK2 and phosphorylated H2AX (γ‐H2AX) [49]. γ‐H2AX, the Ser139‐phosphorylated form of histone H2AX, serves as a sensitive marker of DNA damage and facilitates the recruitment of DNA repair factors and chromatin remodelling complexes [50]. Resection of the 5′ ends of the DSBs produces 3′ single‐stranded DNA (ssDNA), which is immediately coated by replication protein A (RPA) to prevent secondary structure formation. With the assistance of breast cancer type 1 susceptibility protein (BRCA1), partner and localizer of BRCA2 (PALB2) and breast cancer type 2 susceptibility protein (BRCA2), RPA is subsequently replaced by RAD51 Recombinase, forming a RAD51‐ssDNA nucleoprotein filament. This filament invades the homologous region of the sister chromatid to form a displacement loop (D‐loop), enabling template‐directed DNA synthesis and strand resolution, ultimately restoring genomic integrity and allowing cell cycle progression [12, 51].

The NHEJ pathway is initiated by the binding of the Ku70/Ku80 heterodimer to the DSB ends, which stabilises the DNA termini and recruits the catalytic subunit of DNA‐dependent protein kinase (DNA‐PKcs), forming the DNA‐PK complex. Through autophosphorylation and substrate phosphorylation, DNA‐PK promotes synapsis formation and facilitates end processing and ligation, completing the repair process [52].

ncRNAs, including miRNAs and lncRNAs, have been shown to influence radioresistance by modulating DNA repair capacity. For example, miR‐22 enhances radiosensitivity by negatively regulating Sirtuin 1 (SIRT1), leading to increased γ‐H2AX expression and improved DNA damage recognition [30] (Figure 2, Table 1). Similarly, miR‐155 directly targets the 3′‐UTR of RAD51, downregulating its expression and impairing HR efficiency, thereby sensitising breast cancer cells to IR [31] (Figure 2, Table 1). miR‐205 simultaneously targets ZEB1 (Zinc finger E‐box binding homeobox 1, a transcription factor involved in epithelial‐to‐mesenchymal transition) and Ubc13 (Ubiquitin‐conjugating enzyme E2 N, an enzyme involved in HR repair), suppressing DNA repair pathways to enhance radiotherapy sensitivity. Studies have shown that under radiation induction, miR‐205 expression decreases while ZEB1 expression increases, leading to the activation of Ubc13 expression and HR repair functions, resulting in radiotherapy resistance. Overexpression of miR‐205 can disrupt this pathway, inhibit DNA repair and increase cell sensitivity to IR [32] (Figure 2, Table 1).

**FIGURE 3:**
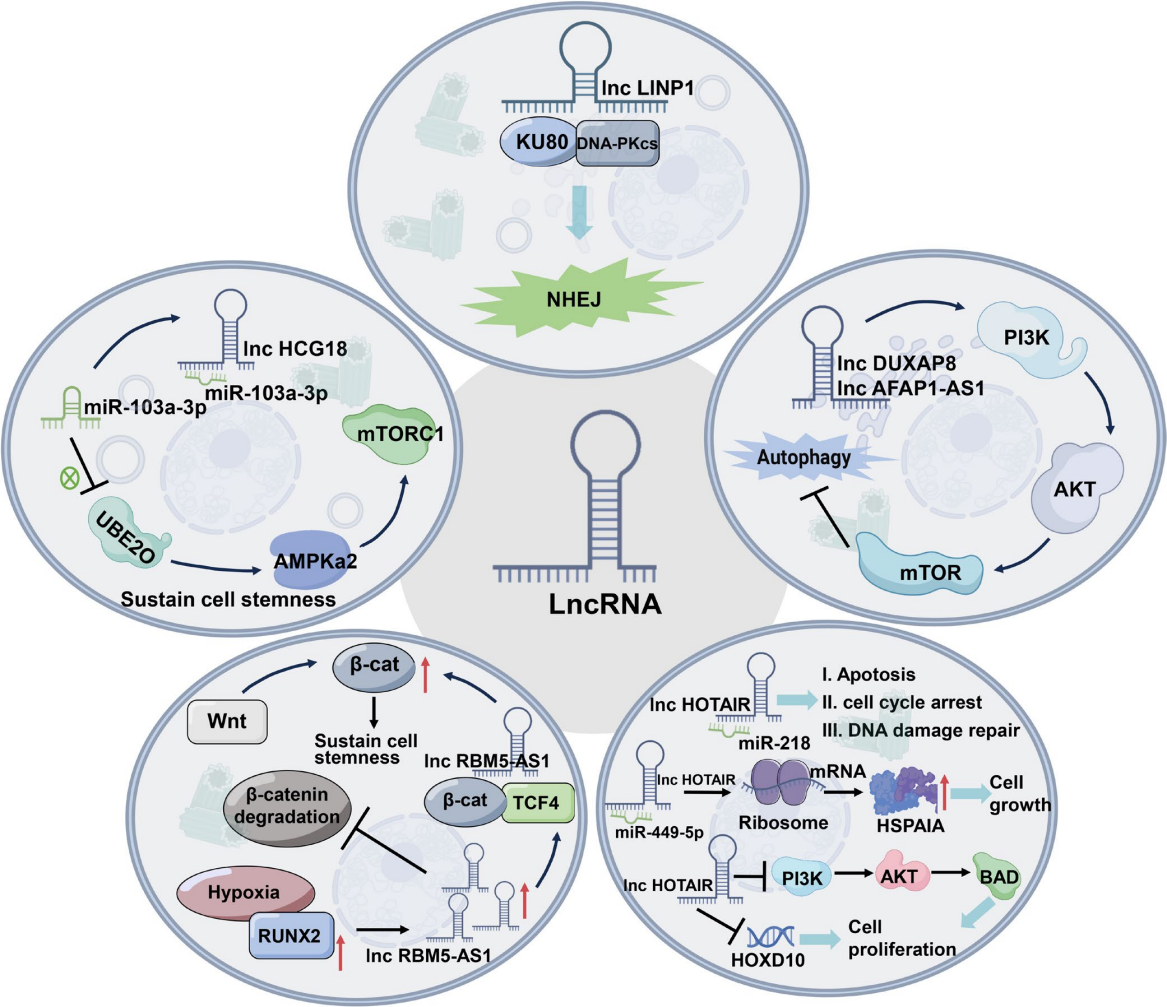
The primary mechanisms by which lncRNAs influence radioresistance in breast cancer cells. (1) The lncRNA LINP1 acts as a scaffold connecting Ku80 and DNA‐PKcs and promotes effective DSBs repair through NHEJ, thereby enhancing the radioresistance of BC cells. (2) lncRNA DUXAP8 and lncRNA AFAP1‐AS1 activate the PI3K/AKT/mTOR pathway to inhibit autophagy and enhance the radioresistance of breast cancer cells. (3) lncRNA HCG18 functions as a sponge for miR‐103a‐3p, positively regulating the expression of the breast cancer‐related ubiquitin‐conjugating enzyme E2O (UBE2O). This regulation enhances the malignant characteristics of breast cancer cells via the UBE2O/AMPKα2/mTORC1 signalling pathway. (4) Hypoxia‐induced Runx family transcription factor 2 (RUNX2) activates the transcription of lncRNA RBM5‐AS1, which inhibits the degradation of β‐catenin. This stabilisation of the β‐catenin‐TCF4 transcriptional complex activates Wnt/β‐catenin signalling, contributing to the maintenance of stemness in breast cancer cells. (5) lncRNA HOTAIR functions as a sponge for miR‐218, reducing radiation‐induced apoptosis, cell cycle arrest, and DNA damage, thereby contributing to radioresistance in breast cancer. Additionally, HOTAIR acts as a competing endogenous RNA (ceRNA) for miR‐449b‐5p, enhancing the expression of HSPA1A. This interaction supports the growth of breast cancer cells under radiation stress, further contributing to radiation resistance. HOTAIR increases radiation resistance by disrupting the activation of the HOXD10 and PI3K/AKT‐BAD signalling pathways.

lncRNAs are also critically involved in DNA repair regulation. lncRNA LINP1 acts as a molecular scaffold linking Ku80 and DNA‐PKcs, thereby promoting NHEJ‐mediated DSB repair. Its expression is regulated by both the p53 and epidermal growth factor receptor (EGFR) signalling pathways, and silencing LINP1 has been shown to enhance the radiosensitivity of triple‐negative breast cancer (TNBC) cells [53] (Figure 3, Table 2). In contrast, the tumour‐suppressive lncRNA GAS5 is frequently downregulated in breast cancer. Overexpression of GAS5 induces G2/M phase arrest and promotes the accumulation of unrepaired DNA damage, ultimately enhancing the cytotoxic effects of radiotherapy [54] (Table 2). Furthermore, lncRNA HOTAIR (HOX transcript antisense RNA) binds to EZH2, a core component of the Polycomb Repressive Complex 2 (PRC2), promoting the recruitment of EZH2 to the MYC promoter region. This enhances its transcriptional activity and subsequently upregulates various NHEJ repair‐related factors (such as Ku70, Ku80, DNA‐PKcs and ATM), increasing the resistance of breast cancer cells to radiotherapy [55] (Figure 3, Table 2).

**TABLE 2:** The roles of specific lncRNAs in regulating radiosensitivity and radioresistance of BC are examined. Each lncRNA, along with its target molecule and its function in the radiation response, is detailed.

lncRNAs	Target	Notes	References
*lncRNA LINP1*	NHEJ pathway	lncRNA LINP1 promotes dsDNA break repair, and inhibiting LINP1 increases tumour cell sensitivity to radiotherapy by modulating the NHEJ pathway	[53]
lncRNA GAS5	miR‐21	lncRNA GAS5 increases G2/M cell cycle arrest and DNA damage, sensitises BC cells to IR by suppressing miR‐21	[54]
*lncRNA HOTAIR*	EZH2	HOTAIR enhances DNA repair and increases breast cancer radioresistance through its interaction with EZH2	[55]
	HOXD10, PI3K/AKT‐BAD pathway	HOTAIR enhances radiation resistance by interfering with the activation of the HOXD10 and PI3K/AKT‐BAD pathway	[56]
*lncRNA FGD5‐AS1*	miR‐497‐5p/MACC1 axis	FGD5‐AS1 increases the radioresistance of BC cells by upregulating MACC1 expression through sponging miR‐497‐5p	[57]
*lncRNA PCAT6*	miR‐185‐5p/TPD52 axis	Silencing PCAT6 enhances the radiosensitivity of TNBC cells by modulating the miR‐185‐5p/TPD52 axis	[58]
*lncRNA CCTA1*	miR‐148b	Downregulation of CCAT1 enhances radiosensitivity through competitive regulation of miR‐148b in breast cancer	[59]
*lncRNA LINC00511*	miR‐185/STXBP4 axis	Silencing LINC00511 enhances radiosensitivity by modulating STXBP4 expression through miR‐185	[60]
*lncRNA LINC00504*	CPEB2	LINC00504 decreases the radiosensitivity of BC cells by binding to TAF15, which stabilises CPEB2 expression	[61]
*lncRNA LINC00963*	FOSB/UBE3C/TP73 axis	LINC00963 enhances the radioresistance of BC cells by the FOSB/UBE3C/TP73 axis	[62]
*lncRNA DUXAP8*	PI3K/ATK/mTOR pathway, EZH2‐E‐cadherin/RHOB axis	lncRNA DUXAP8 enhances the radioresistance of BC cells by activating the PI3K/AKT/mTOR pathway and EZH2‐E‐cadherin/RHOB axis	[63]
*NEAT1*	NQO1	lncRNA NEAT1 increased the radioresistance of TNBC by positively regulating NQO1 expression and CSC activity	[64]
*lncRNA AFAP1‐AS1*	Wnt/β‐catenin pathway	AFAP1‐AS1 induces radioresistance in TNBC by activating the Wnt/β‐catenin signalling pathway	[65]

*Note*: Italics indicate lncRNAs that enhance radioresistance, while underlined lncRNAs are those that promote radiosensitivity.

In summary, breast cancer cells utilise HR and NHEJ pathways to efficiently repair radiation‐induced DSBs and evade the cytotoxicity of IR. ncRNAs modulate this process through complex regulatory networks, acting on key repair molecules to influence cellular radiosensitivity. Targeting these ncRNA‐mediated pathways offers promising strategies to overcome therapeutic resistance and improve radiotherapy outcomes.

#### 1.1.4 Tumour Cells Develop Radioresistance by Modulating Apoptotic Responses

In normal cells, severe DNA damage—such as radiation‐induced DSBs—typically activates intrinsic apoptotic pathways to eliminate genetically unstable cells and prevent malignant transformation. However, tumour cells often evade this programmed cell death by blocking or bypassing apoptotic signalling pathways. This phenomenon, known as apoptotic evasion, is one of the recognised hallmarks of cancer and plays a key role in immune escape and resistance to anticancer therapies [66].

Among various cancer types, the phosphoinositide 3‐kinase/protein kinase B (PI3K/AKT) signalling pathway has been identified as a central regulator of cell survival and apoptosis. This pathway suppresses apoptosis and promotes proliferation by modulating multiple downstream effectors [67]. Specifically, radiation‐induced DSBs activate ATM and ATR kinases. ATM indirectly activates receptor tyrosine kinases (RTKs) and recruits PI3K to the plasma membrane, where PI3K catalyses the conversion of phosphatidylinositol‐4,5‐bisphosphate (PIP2) into phosphatidylinositol‐3,4,5‐trisphosphate (PIP3). This lipid second messenger facilitates the membrane localisation and phosphorylation‐dependent activation of AKT [68, 69]. Activated AKT inhibits apoptosis through multiple mechanisms: (i) It phosphorylates and inactivates the pro‐apoptotic protein BAD (Bcl‐2‐associated death promoter), promoting its sequestration by 14‐3‐3 proteins and preventing mitochondrial outer membrane permeabilisation. (ii) It upregulates the expression of anti‐apoptotic proteins such as Bcl‐2 and Bcl‐xL (Bcl‐2‐like protein 1), stabilising mitochondrial integrity and blocking cytochrome c release. It suppresses the activation of caspase‐9, a key initiator caspase in the mitochondrial apoptotic pathway, thereby inhibiting apoptotic signal cascades. (iii) It inhibits FOXO family pro‐apoptotic transcription factors, reducing the transcription of genes such as Bim (Bcl‐2‐interacting mediator of cell death), FasL (Fas Ligand, also known as CD95L or TNFSF6) and TRAIL (TNF‐related apoptosis‐inducing ligand) that are involved in cell death execution [70, 71, 72].

Recent studies have increasingly highlighted the role of ncRNAs in modulating apoptotic responses and influencing radiosensitivity in tumour cells. For instance, miR‐944 has been shown to suppress the PI3K/AKT pathway and downregulate SPP1 (Secreted Phosphoprotein 1, also known as Osteopontin), thereby inhibiting proliferation and promoting apoptosis in breast cancer cells. SPP1 is a secreted glycoprotein involved in tumour growth, survival and metastasis, and its expression is closely associated with therapeutic resistance [33] (Table 1). Additionally, overexpression of miR‐200c can downregulate phosphorylated epidermal growth factor receptor (p‐EGFR), inhibiting the PI3K/AKT and extracellular signal‐regulated kinase (ERK) pathways. This enhances radiation‐induced apoptosis and prolongs the duration of γH2AX foci, indicating an increase in DSB damage induced by radiotherapy [27] (Figure 2, Table 1). miR‐200c can also target TANK‐binding kinase 1 (TBK1), reducing its pro‐survival function. TBK1 is a serine/threonine kinase that regulates the activity of NF‐κB and AKT pathways, exhibiting anti‐apoptotic functions in various tumours [28] (Figure 2, Table 1). Another key miRNA is the let‐7 family, which targets breast cancer stem cells (CSCs) and regulates the Cyclin D1/AKT1/Wnt1 signalling pathway, significantly enhancing radiotherapy sensitivity [34] (Figure 2, Table 1). In contrast, the lncRNA HOTAIR enhances breast cancer cell radioresistance by activating the HOXD10/PI3K/AKT/BAD pathway [56] (Figure 2, Table 2). miR‐95 activates the PI3K/AKT pathway and inhibits apoptosis by targeting sphingosine‐1‐phosphate phosphatase 1 (SGPP1), thereby increasing radiotherapy resistance in various tumours [35] (Figure 2, Table 1).

Moreover, PTEN (Phosphatase and Tensin Homologue deleted on chromosome 10) is a critical negative regulator of the PI3K/AKT pathway. As a lipid phosphatase, PTEN dephosphorylates PIP3 back to PIP2, thereby blocking AKT activation and terminating downstream anti‐apoptotic signalling. Several breast cancer‐associated ncRNAs, such as miR‐221/222 and piR‐651, have been reported to downregulate PTEN, consequently enhancing AKT signalling and contributing to radioresistance. In contrast, upregulating PTEN expression can suppress AKT activity, enhance radiation‐induced apoptosis and restore radiosensitivity in tumour cells [36, 73] (Figure 2, Table 1).

Additionally, several ncRNAs regulate other pro‐apoptotic and anti‐apoptotic factors, influencing the response to radiotherapy: lncRNA FGD5 antisense transcript 1 (FGD5‐AS1) acts as a sponge for miR‐497‐5p, relieving its inhibition on the metastasis‐associated in colon cancer 1 (MACC1) gene, thereby promoting radiotherapy resistance [57] (Figure 2, Table 1). Prostate cancer‐associated transcript 6 (PCAT6) upregulates the expression of tumour protein D52 (TPD52), reducing apoptosis in tumour cells [58] (Table 2). lncRNA CCAT1 (colon cancer‐associated transcript 1) promotes apoptosis and enhances radiotherapy sensitivity by regulating miR‐148b [59] (Table 2). LINC00511 reduces apoptosis by regulating the miR‐185/STXBP4 axis [60], while LINC00504 interacts with TAF15 to stabilise CPEB2 expression, diminishing radiotherapy sensitivity [61] (Table 2). lncRNA LINC00963 promotes the nuclear translocation of the transcription factor FOSB, enhancing the transcriptional activity of ubiquitin protein ligase 3C (UBE3C), which induces the degradation of tumour protein p73 (TP73) and weakens its pro‐apoptotic function [62] (Table 2).

**FIGURE 4:**
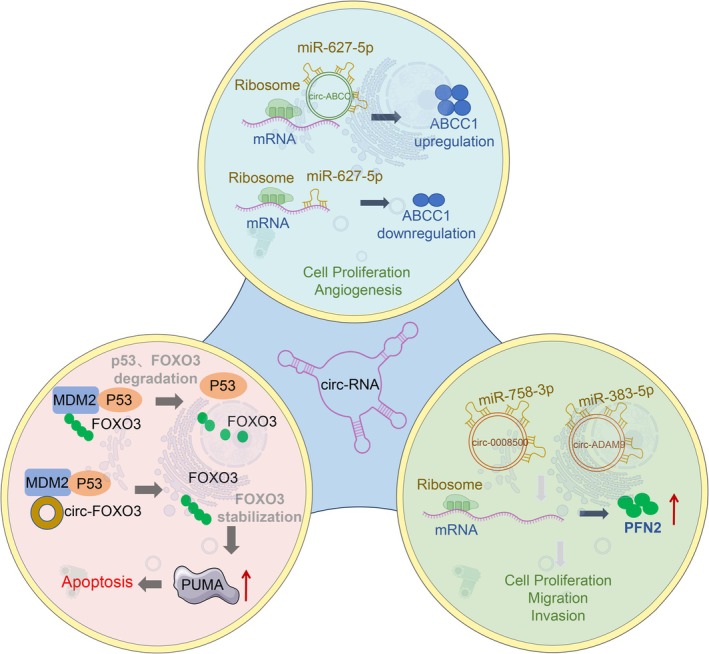
The primary mechanisms by which circRNAs influence radioresistance in breast cancer cells. (1) MDM2 is an E3 ubiquitin ligase that controls the ubiquitination and degradation of p53 and Foxo3 in the proteasome. circ‐Foxo3 promotes the formation of the p53‐MDM2 complex, enhancing the poly‐ubiquitination and degradation of p53, while simultaneously reducing MDM2‐mediated degradation of Foxo3. As a result, Foxo3 protein accumulates, leading to the upregulation of the apoptotic gene PUMA and increasing cell apoptosis. (2) ABCC1 is part of the ATP‐binding cassette (ABC) transporter family and plays a role in the proliferation and angiogenesis of breast cancer cells. circ‐ABCC1 increases the expression of its host gene ABCC1 by competitively binding to miR‐627‐5p, which promotes the resistance of breast cancer cells to radiotherapy. (3) The profilin 2 (PFN2) is a member of the actin‐binding protein family, and its overexpression promotes the proliferation, migration, invasion and EMT of TNBC cells. circ_0008500 and circ‐ADAM9 enhance breast cancer radioresistance by targeting miR‐758‐3p and miR‐383‐5p to reduce PFN2 expression.

In the realm of circRNAs, circ‐Foxo3 is a circRNA formed by the reverse splicing of the Forkhead box O3 (FOXO3) gene, primarily localised in the cytoplasm. As one of the more thoroughly researched cancer‐related circRNAs, circ‐Foxo3 can form stable complexes with various circular RNA‐binding proteins (cRBPs), playing a key regulatory role in tumour biology [74]. Studies have shown that circ‐Foxo3 exhibits significant tumour suppressor functions in breast cancer, with its ectopic expression inducing cellular stress responses and activating apoptotic programmes, thereby inhibiting tumour cell growth. In breast cancer cells, circ‐Foxo3 is typically expressed at low levels; however, under conditions that induce apoptosis, its expression is significantly upregulated, suggesting a crucial role in stress‐induced cell death. Mechanistically, circ‐Foxo3 promotes the formation of a complex between mouse double minute 2 homologue (MDM2) and the tumour suppressor protein p53, enhancing p53's ubiquitination and degradation. MDM2 is an E3 ubiquitin ligase that primarily negatively regulates apoptotic signals by modulating the ubiquitination of p53 and FOXO3. However, the interaction between circ‐Foxo3 and MDM2 facilitates p53 degradation while simultaneously hindering MDM2's ubiquitination of FOXO3, thereby protecting FOXO3 from degradation. The accumulation of FOXO3 further upregulates the expression of its target gene, PUMA (p53 upregulated modulator of apoptosis), activating the mitochondrial pathway‐mediated apoptotic response and ultimately enhancing breast cancer cell sensitivity to apoptotic stimuli. In summary, circ‐Foxo3 plays a significant tumour‐suppressive role in regulating apoptosis and radiotherapy sensitivity in breast cancer by modulating the MDM2‐p53‐FOXO3 axis [75] (Figure 4).

In summary, breast cancer cells gain radioresistance by activating the PI3K/AKT pathway and modulating a range of apoptosis‐related molecules to evade cell death. ncRNAs, by regulating components of this pathway, serve as critical modulators of apoptotic responses and represent promising therapeutic targets to overcome resistance to radiotherapy.

#### 1.1.5 Tumour Cells Confer Radioresistance via the Regulation of Autophagy

Autophagy is a lysosome‐mediated process responsible for the degradation and recycling of damaged organelles, misfolded proteins and intracellular pathogens, thereby maintaining cellular homeostasis [76]. Recent studies have highlighted its critical role in tumour development, therapeutic resistance and response to radiotherapy [77].

Under conditions of nutrient deprivation or cellular stress—such as hypoxia, radiation, or oxidative stress—AMP‐activated protein kinase (AMPK) is activated and inhibits the mammalian target of rapamycin (mTOR) pathway, thereby relieving mTOR‐mediated suppression of autophagy initiation [78]. Concurrently, stress stimuli can promote the translocation of High Mobility Group Box 1 (HMGB1) from the nucleus to the cytoplasm. Cytoplasmic HMGB1 facilitates autophagy by binding directly to Beclin‐1 (encoded by BECN1), promoting its release from inhibitory interactions with anti‐autophagic proteins such as Bcl‐2. Additionally, HMGB1 can further suppress mTOR activity by modulating the PI3K/AKT/mTOR signalling pathway, synergistically enhancing autophagy through multiple mechanisms [79, 80].

In this regulatory network, the activation of the Unc‐51‐like autophagy activating kinase 1 (ULK1) complex marks the completion of the autophagy initiation stage [81]. Subsequently, Beclin‐1 associates with Vacuolar Protein Sorting 34 (VPS34)—a class III phosphatidylinositol 3‐kinase—to form the PI3KC3 complex, which initiates the formation of the isolation membrane (phagophore) [80, 82]. Members of the ATG (autophagy‐related) protein family, such as ATG5, ATG12 and ATG16L1, facilitate phagophore elongation and the lipidation of LC3. Cytosolic LC3‐I (microtubule‐associated protein 1A/1B‐light chain 3‐I) is conjugated to phosphatidylethanolamine by ATG7 and ATG3, forming LC3‐II, which is inserted into the autophagosome membrane and serves as a hallmark of autophagic activity [83]. Mature autophagosomes subsequently fuse with lysosomes to form autolysosomes, a process dependent on Rab7 (Ras‐related protein Rab‐7) and the SNARE (soluble N‐ethylmaleimide‐sensitive factor attachment protein receptor) complex [84]. Within the autolysosome, lysosomal hydrolases such as Cathepsins degrade the sequestered cytoplasmic cargo. The resulting metabolic products—including amino acids, fatty acids and sugars—are recycled by the cell to maintain energy homeostasis and survival under stress conditions like radiation exposure [85, 86].

A growing body of evidence indicates that tumour cells can acquire radioresistance by modulating autophagy through the above mechanisms. Notably, ncRNAs play critical regulatory roles in autophagy‐related pathways. For instance, miR‐129‐5p has been shown to sensitise breast cancer cells to radiation by targeting and downregulating HMGB1, thereby suppressing autophagy and reducing post‐irradiation survival [37] (Figure 2, Table 1). Additionally, miR‐200c targets Ubiquilin 1 (UBQLN1), inhibiting the radiation‐induced autophagy process, thereby further increasing the sensitivity of breast cancer cells to radiotherapy. UBQLN1 is a multifunctional protein containing ubiquitin‐like and ubiquitin‐binding domains, primarily involved in regulating the ubiquitin‐proteasome system. It plays a critical role in autophagy by assisting in the recognition and transport of ubiquitinated substrates to autophagosomes and facilitating the fusion of autophagosomes with lysosomes, thereby maintaining intracellular protein homeostasis [29] (Figure 2, Table 1). In contrast, Lei et al. reported that the lncRNA DUXAP8 is upregulated in radioresistant breast cancer tissues and enhances autophagic activity via activation of the PI3K/AKT/mTOR pathway, thereby promoting resistance to radiation [63] (Figure 3, Table 2).

In conclusion, autophagy acts as a key cytoprotective mechanism in response to radiotherapy‐induced stress in tumour cells, and its regulatory network is highly complex. Elucidating the molecular interplay between autophagy and radioresistance—particularly the role of ncRNAs—may provide novel strategies to enhance the efficacy of radiotherapy and overcome resistance in breast cancer.

#### 1.1.6 Tumour Cells Confer Radioresistance by Modulating Oxidative Stress Responses

ROS, including superoxide anion (O_2_^−^), hydrogen peroxide (H_2_O_2_), and hydroxyl radicals (•OH), are highly reactive, short‐lived molecules that function as byproducts of cellular metabolism. Initially identified in skeletal muscle, ROS is ubiquitously present across diverse cell types [87]. During radiotherapy, IR induces the radiolysis of water molecules, leading to the generation of abundant ROS. These species subsequently inflict various forms of DNA damage, including DSBs and base oxidation [17]. Notably, approximately 80% of radiation‐induced DNA lesions are mediated by ROS, underscoring their critical role as both executors of radiation‐induced cytotoxicity and major contributors to intracellular oxidative stress [16].

In the context of breast cancer radioresistance, tumour cells exhibit a high degree of adaptability to oxidative stress, enabling them to counteract ROS‐mediated damage and survive radiotherapeutic insult. To mitigate ROS‐induced cytotoxicity, breast cancer cells activate endogenous antioxidant defence systems that effectively scavenge ROS, thereby attenuating oxidative stress and enhancing radiotolerance. This antioxidant network involves the coordinated activity of key enzymes, including superoxide dismutase (SOD), catalase (CAT) and glutathione peroxidase (GPx). SOD catalyses the dismutation of superoxide anions into H_2_O_2_, which is subsequently converted into water and oxygen by CAT and GPx. This enzymatic cascade efficiently neutralises intracellular ROS and protects vital biomolecules such as DNA, proteins and lipid membranes from oxidative damage [88, 89].

Mechanistically, breast cancer cells have been shown to significantly upregulate SOD and GPx expression following radiation exposure. This stress‐adaptive response facilitates the detoxification of radiation‐induced ROS, thereby promoting cell survival and contributing to radioresistance. Moreover, recent studies have demonstrated that miR‐139‐5p enhances radiosensitivity by targeting multiple genes involved in ROS detoxification, highlighting its potential therapeutic value in overcoming radioresistance [38] (Figure 2, Table 1).

Collectively, these findings suggest that breast cancer cells can evade radiation‐induced cytotoxicity by augmenting their antioxidant capacity. Targeting the associated antioxidant pathways—particularly through the regulatory actions of ncRNAs—may represent a promising strategy to enhance the efficacy of radiotherapy in breast cancer.

#### 1.1.7 Tumour Cells Promote Radiotherapy Resistance Through Remodelling of the TME

The TME plays a pivotal role in modulating the response of tumour cells to radiotherapy [90]. Cancer‐associated fibroblasts (CAFs), recognised as key “architectural organisers” within the TME, facilitate tumour progression and metastasis by secreting various cytokines and remodelling the extracellular matrix (ECM) [91]. Notably, following radiotherapy, CAFs exhibit high radiation tolerance, often surviving high‐dose exposure and entering a state of senescence, during which they secrete numerous immunomodulatory factors. These secretions impair T cell‐mediated anti‐tumour immunity and enhance immune evasion, thereby diminishing the therapeutic efficacy of radiotherapy and contributing to resistance [92, 93].

In addition to CAFs, immune cells within the TME are critically involved in radiotherapy resistance. Tumour tissues frequently exhibit an immunosuppressive landscape, wherein suppressive immune populations—such as tumour‐associated macrophages (TAMs) and regulatory T cells (Tregs)—outnumber effector immune cells. These suppressive cells release inhibitory cytokines that dampen effector T cell function, consequently enhancing tumour cell survival and radioresistance [94, 95, 96].

Angiogenesis and hypoxia within the TME further modulate tumour radiosensitivity. Hypoxic conditions activate the hypoxia‐inducible factor‐1 (HIF‐1) signalling cascade, promoting neovascularisation and improving tumour nutrient supply and survival [97]. Moreover, HIF‐1 activation facilitates metabolic reprogramming and augments DNA damage repair capacity, collectively contributing to increased resistance to radiation [98, 99].

At the molecular level, a growing body of evidence highlights the role of ncRNAs as key regulators of TME‐mediated radioresistance. For instance, circular RNA TBPL1 (circTBPL1) is upregulated in exosomes derived from breast cancer‐associated CAFs. Functionally, circTBPL1 acts as a molecular “sponge” for microRNA‐653‐5p, thereby protecting trophoblast glycoprotein (TPBG)—a highly glycosylated transmembrane protein implicated in tumour proliferation, invasion and metastasis—from degradation, ultimately promoting breast cancer cell growth and dissemination [100].

Similarly, the lncRNA small nucleolar RNA host gene 5 (SNHG5) is highly expressed in breast cancer CAFs, where it facilitates angiogenesis and vascular permeability by regulating the expression of zinc finger protein 281 (ZNF281), contributing to the formation of the pre‐metastatic niche (PMN) [101]. Other studies have demonstrated that certain ncRNAs enhance TAM‐mediated radioresistance by promoting M2 macrophage polarisation [102]. Additionally, breast cancer cells can activate the TGF‐β signalling pathway to upregulate miR‐182 in TAMs, which directly targets Toll‐like receptor 4 (TLR4), driving TAMs toward an M2 phenotype and accelerating tumour progression [103]. lncRNA SNHG1 has also been implicated in promoting M2 macrophage polarisation, thereby supporting breast cancer cell proliferation and angiogenesis [104].

ATP‐binding cassette sub‐family C member 1 (ABCC1) belongs to the ATP‐binding cassette (ABC) superfamily and is an important membrane transporter that relies on ATP hydrolysis to drive the transmembrane transport of substrates. ABCC1 is aberrantly expressed in various tumours, and its functions involve cellular drug efflux, metabolite transport and cell signalling regulation, thereby participating in key biological processes such as tumour cell proliferation, angiogenesis and chemotherapy resistance. In breast cancer, ABCC1 mediates the efflux of chemotherapy drugs through a drug pump mechanism, increasing cellular resistance. Additionally, it regulates cell communication and signalling in the TME, promoting angiogenesis and supporting tumour growth and metastasis [105, 106, 107]. In recent years, circRNA circ‐ABCC1 has been found to regulate the expression level of its host gene ABCC1. circ‐ABCC1 acts as a “miRNA sponge” by competitively binding to microRNA‐627‐5p (miR‐627‐5p), inhibiting its targeted suppression of ABCC1, which results in a significant upregulation of ABCC1 expression. The high expression of ABCC1 further enhances the multidrug resistance and radioresistance of breast cancer cells, promoting tumour cell survival under radiotherapy stress [41] (Figure 4, Table 3).

**TABLE 3:** The roles of specific circRNAs in regulating radiosensitivity and radioresistance of BC are examined. Each circRNA, along with its target molecule and its function in the radiation response, is detailed.

circRNAs	Target	Notes	References
*circNCOR1*	hsa‐miR‐638	circNCOR1 regulates the expression of CDK2 by sponging hsa‐miR‐638 and reduces the radiosensitivity of TNBC cells	[40]
*circ‐ABCC1*	miR‐627‐5p	circ‐ABCC1 increases the expression of its host gene ABCC1 by sponging miR‐627‐5p, which promotes radioresistance of BC cells	[41]
*circ_0008500*	miR‐758‐3p	Downregulated circ_0008500 increases BC radiosensitivity by targeting miR‐758‐3p to lower PFN2 expression	[42]
*circ‐ADAM9*	miR‐383‐5p/PFN2 axis	circ‐ADAM9 downregulation enhances breast cancer radiosensitivity via the miR‐383‐5p/PFN2 axis	[43]

*Note*: Italics indicate circRNAs that enhance radioresistance, while underlined circRNAs are those that promote radiosensitivity.

Under hypoxic conditions, HIF‐1α suppresses the expression of miR‐338‐3p, relieving its inhibitory effect on zinc finger E‐box‐binding homeobox 2 (ZEB2), a key transcription factor driving epithelial–mesenchymal transition (EMT), migration and invasion. This axis is coordinated by the activation of NF‐κB and PI3K/Akt signalling pathways [108] (Figure 2). Furthermore, HIF‐1α promotes transcriptional activation of lncRNA urothelial cancer‐associated 1 (UCA1). Silencing UCA1 significantly suppresses breast cancer cell proliferation and induces apoptosis under hypoxic conditions, underscoring its critical role in radioresistance [109].

Collectively, these findings underscore that tumour cells orchestrate a complex and coordinated remodelling of the TME—including fibroblasts, immune cells, hypoxic responses and ncRNA regulators—to establish systemic radioresistance. Targeting these TME‐driven resistance pathways, particularly those modulated by ncRNAs, may offer promising therapeutic avenues for enhancing the radiosensitivity of breast cancer.

#### 1.1.8 Tumour Cells Mediate Radioresistance Through Regulation of Stemness

CSCs represent a subpopulation of tumour cells endowed with self‐renewal capacity and multipotent differentiation potential [110]. These cells typically reside in hypoxic, acidic (low pH) and nutrient‐deprived niches within the TME, where they exhibit pronounced intratumoral heterogeneity, potent proliferative capacity and marked resistance to conventional therapies. These characteristics collectively confer a pivotal role for CSCs in tumour initiation, progression, metastasis and radioresistance [111].

CSCs mediate radioresistance through multiple, interconnected mechanisms. They orchestrate EMT, a process essential for enhanced invasiveness and dissemination, and exhibit elevated expression of drug efflux transporters as well as potent ROS‐scavenging capabilities that promote cellular survival under genotoxic stress. Additionally, CSCs display protective autophagy and phenotypic plasticity, enabling dynamic interconversion between stem‐like (EMT phenotype) and non‐stem‐like (mesenchymal–epithelial transition, MET phenotype) states, as well as transitions between quiescent (therapy‐resistant) and proliferative (therapy‐sensitive) states. Their intrinsic anti‐apoptotic properties and efficient DNA damage repair systems further enhance resistance to radiotherapy [112].

Hypoxia, a hallmark of the TME, plays a central role in promoting CSC‐associated radioresistance. Oxygen deprivation enhances CSC heterogeneity and adaptability, potentially establishing a positive feedback loop that sustains therapeutic resistance. For instance, under hypoxic conditions, miR‐210 is upregulated in breast cancer CSCs and facilitates stemness, migration, proliferation and self‐renewal via direct targeting of E‐cadherin [113] (Figure 2).

Conversely, miR‐142‐3p is frequently downregulated in breast cancer and acts as a radiosensitiser by suppressing β‐catenin, thereby attenuating CSC properties and increasing radiotherapy responsiveness [39] (Figure 2, Table 1).

Emerging evidence has also revealed the regulatory role of lncRNAs in maintaining CSC phenotypes and modulating radioresistance. For example, HCG18 (HLA complex group 18) is upregulated in breast cancer tissues and cell lines, where it promotes tumour cell proliferation, invasion and stemness. Mechanistically, HCG18 acts as a ceRNA for miR‐103a‐3p, thereby derepressing UBE2O (ubiquitin‐conjugating enzyme E2O) and activating the AMPKα2/mTORC1 signalling axis, ultimately reinforcing malignant phenotypes [114] (Figure 3).

Another lncRNA, NEAT1 (nuclear paraspeckle assembly transcript 1), has been shown to positively regulate CSC activity. Silencing NEAT1 significantly enhances the radiosensitivity of TNBC cells, underscoring its potential as a therapeutic target to overcome radioresistance [64] (Table 2).

Profilin 2 (PFN2), a member of the actin‐binding protein family, is significantly overexpressed in TNBC tissues. As a crucial regulator of the cytoskeleton, PFN2 primarily influences cell morphology, motility and signalling by regulating the dynamics of actin polymerisation. Research indicates that the overexpression of PFN2 enhances the proliferation, migration and invasiveness of TNBC cells by promoting EMT, thereby driving malignant progression and metastasis. The EMT process not only endows cancer cells with greater invasive potential but is also closely associated with CSC characteristics, serving as a significant mechanism of tumour radioresistance [115]. At the level of ncRNA regulation, circRNA circ_0008500 has been found to effectively regulate PFN2 expression by competitively binding to microRNA‐758‐3p (miR‐758‐3p). Specifically, circ_0008500 acts as a “sponge” for miR‐758‐3p, blocking its inhibitory effect on PFN2, leading to sustained high levels of PFN2 expression and promoting EMT and radioresistance in tumour cells [42] (Figure 4, Table 3). A similar mechanism is observed with another circRNA, circ‐ADAM9, which regulates PFN2 expression by targeting the miR‐383‐5p/PFN2 axis, affecting tumour cell sensitivity to radiotherapy [43] (Figure 4, Table 3).

In summary, CSCs possess both intrinsic and adaptive mechanisms that collectively contribute to radiotherapy resistance, including enhanced DNA repair, ROS detoxification, EMT plasticity and activation of diverse survival pathways. These traits are tightly modulated by hypoxic stress and ncRNAs, highlighting the CSC–ncRNA axis as a critical mediator of radioresistance in breast cancer and a promising target for therapeutic intervention.

#### 1.1.9 Tumour Cells Mediate Radioresistance by Regulating Ferroptosis

Ferroptosis is a form of programmed cell death characterised by iron‐dependent accumulation of lipid peroxides. The core mechanism involves intracellular iron catalysing ROS generation via the Fenton reaction, which drives lipid peroxidation of polyunsaturated fatty acids (PUFAs). Excessive accumulation of lipid peroxides on cellular membranes, coupled with impaired clearance, leads to membrane damage and subsequent cell death. Glutathione peroxidase 4 (GPX4) serves as a critical negative regulator of ferroptosis by reducing lipid peroxides to non‐toxic lipid alcohols in a glutathione (GSH)‐dependent manner. Depletion of GSH or inactivation of GPX4 results in lipid peroxide accumulation and triggers ferroptosis [116]. Tumour cells can manipulate this pathway to reduce sensitivity to radiation‐induced oxidative stress, thereby conferring radioresistance [117].

Recent studies have unveiled the pivotal roles of ncRNAs in regulating ferroptosis and tumour progression. For instance, miR‐324‐3p directly targets the 3′‐UTR of GPX4 mRNA, downregulating its expression to enhance ferroptosis and suppress breast cancer cell viability [118]. Conversely, the lncRNA RUNX1 intronic transcript 1 (RUNX1‐IT1), highly expressed in breast cancer, interacts with the N6‐methyladenosine (m6A) reader protein IGF2BP1 (insulin‐like growth factor 2 mRNA‐binding protein 1), promoting liquid–liquid phase separation (LLPS) that stabilises GPX4 mRNA and inhibits ferroptosis, thus facilitating radioresistance [119, 120].

Beyond ncRNAs, various tumour suppressors promote ferroptosis to exert anti‐tumour effects. Acetylation of specific lysine residues on p53 represses transcription of SLC7A11 (solute carrier family 7 member 11), the key subunit of system Xc^−^ antiporter responsible for cystine uptake in exchange for glutamate. By limiting cystine import, SLC7A11 downregulation reduces intracellular cysteine availability, depleting GSH synthesis, impairing GPX4 activity and ultimately inducing ferroptosis [121, 122]. Notably, the lncRNA P53RRA, downregulated in breast cancer, binds to G3BP1 (Ras GTPase‐activating protein‐binding protein 1), a multifunctional RNA‐binding protein involved in stress granule assembly and mRNA metabolism, facilitating nuclear retention and accumulation of p53. This interaction induces cell cycle arrest, apoptosis and ferroptosis [123].

Ferroptosis is also negatively regulated by specific factors [124]. AIFM2 (apoptosis‐inducing factor mitochondria‐associated 2), a ubiquinone oxidoreductase, functions as an anti‐ferroptotic factor. In HER2‐positive breast cancer, circRNA GFRA1 is overexpressed and acts as a molecular sponge for miR‐1228, relieving repression of AIFM2 and thereby inhibiting ferroptosis [125]. Similarly, circRHOT1 downregulates ferroptosis via the miR‐106a‐5p/STAT3 signalling axis, promoting breast cancer progression [126].

In summary, ferroptosis represents a critical regulatory node in tumour cell response to radiotherapy. Tumour cells suppress ferroptosis through coordinated regulation of ncRNAs, inhibitory factors and key signalling molecules to achieve radioresistance. Detailed elucidation of ferroptosis regulatory mechanisms and targeting of pivotal nodes holds promise for enhancing radiosensitivity in cancer therapy.

### 1.2 Translational Strategies and Challenges for ncRNA‐Based Radiosensitisation

Various molecular approaches have been developed to modulate ncRNA expressions, aiming to improve radiosensitivity and clinical outcomes. RNA‐based therapeutics currently include antisense oligonucleotides (ASOs), small interfering RNAs (siRNAs), antagomiRs and chemically modified miRNA mimics, which achieve targeted regulation by suppressing or activating specific ncRNAs. Additionally, genome editing technologies such as CRISPR/Cas9 enable precise manipulation of ncRNA expression [127, 128, 129].

Despite rapid progress, clinical translation of RNA therapeutics faces significant hurdles: (i) Immune activation and toxicity: Exogenous RNAs can be recognised by innate immune sensors, triggering inflammatory responses. High doses or sequence homology may cause off‐target toxicities [130, 131]. (ii) Delivery specificity: Achieving selective targeting remains challenging; uptake by non‐target cells leads to off‐target effects [132, 133, 134]. (iii) Intracellular trafficking barriers: Efficient endosomal escape is required for therapeutic efficacy, representing a major bottleneck [135]. (iv) Poor stability and pharmacokinetics: Naked nucleic acids are susceptible to nuclease degradation, exhibit short plasma half‐lives, and are rapidly cleared renally, limiting therapeutic windows [136, 137].

To address these issues, diverse chemical modifications (phosphorothioate backbones, 2′‐O‐methyl or 2′‐fluoro modifications, locked nucleic acids) enhance stability [138, 139]. Delivery strategies employing lipids, cholesterol conjugates, aptamers, antibodies and cell‐penetrating peptides (CPPs) improve targeting [139]. PEGylation and CD47 mimetics prolong circulation time and reduce immune clearance [140].

Nanoparticles (NPs) offer versatile platforms to overcome vascular barriers, endocytosis obstacles and stability concerns due to their tunable structure, biocompatibility and targeting potential [141]. For example, our group identified lncRNA AFAP1‐AS1 as a mediator of radioresistance in TNBC via activation of the Wnt/β‐catenin pathway. We developed redox‐responsive polymeric nanoparticles (PDSA‐NPs) to deliver siAFAP1‐AS1 combined with glutathione depletion, synergistically reversing resistance and significantly enhancing radiotherapy efficacy in vivo [65] (Figure 3, Table 2).

Collectively, ncRNA‐targeted radiosensitisation strategies exhibit promising translational potential. However, clinical application requires overcoming challenges in stability, specificity, delivery efficiency and toxicity control. Future efforts should focus on optimising delivery platforms in conjunction with mechanistic studies to advance ncRNA‐based radiotherapy sensitisers into clinical practice.

## 2 Challenges, Limitations and Future Directions

Despite the promising role of ncRNAs in mediating radioresistance in breast cancer, their clinical translation remains constrained by several key challenges and limitations. Emerging therapeutic strategies—including drug repurposing approaches such as GLP‐1‐based therapies or proteasome‐targeting agents, as well as natural immunomodulators like prodigiosin—may offer complementary avenues to overcome these barriers while enhancing radiosensitivity [90, 142, 143].

Lack of experimental standardisation and high‐quality clinical data. Considerable heterogeneity in experimental methodologies—including control selection, RNA extraction protocols and quantification techniques—undermines the reproducibility and comparability of ncRNA expression data [144]. Moreover, inadequate reporting of clinical outcomes limits the ability to perform robust meta‐analyses assessing the relevance of specific ncRNAs to radiotherapy response [145]. Future investigations should prioritise methodological standardisation and employ large‐scale, multicentre cohorts to identify the most clinically relevant ncRNA candidates associated with radiosensitivity. Rigorous data acquisition is essential to support evidence‐based evaluation of ncRNAs in this context.

Accessibility and reliability of biomarkers. Comparing ncRNA expression profiles in tumour tissue with those in accessible body fluids (e.g., peripheral blood) may enable the identification of non‐invasive biomarkers predictive of radiotherapy response, thereby circumventing the limitations of repeated tissue biopsies. Several studies have reported that circulating ncRNAs—such as miR‐22, miR‐155 and miR‐19a—can predict radiosensitivity in breast cancer [146, 147]. However, high inter‐individual variability in baseline circulating ncRNA levels complicates the development of reliable receiver operating characteristic (ROC) curves [148]. Future efforts should focus on validating multi‐ncRNA signature panels to enhance predictive accuracy, and exploring alternative biofluids (e.g., urine and saliva) may open new avenues for minimally invasive diagnostics.

Target selection and therapeutic strategy development. Although numerous ncRNAs have been identified, only a fraction is functionally validated as mediators of radioresistance, highlighting the need for rigorous target prioritisation. Therapeutic approaches targeting ncRNAs—such as siRNAs, ASOs and CRISPR/Cas9‐based editing—remain in early developmental stages and face technical hurdles including off‐target effects, inefficient delivery and immunogenicity [128]. Future research should aim to improve delivery systems through enhanced chemical modifications and nanoparticle‐based carriers, while advancing our understanding of ncRNA‐centred regulatory networks to avoid unintended biological consequences from excessive pathway perturbation.

Complex regulatory networks and safety concerns. ncRNAs participate in intricate regulatory circuits governing cancer cell behaviour, yet their physiological roles and downstream targets are not fully elucidated. Clinical data on the safety and efficacy of ncRNA‐targeted therapies remain scarce. From target discovery to therapeutic development, and from preclinical validation to clinical translation, ncRNA‐based interventions still face a long developmental trajectory. Moving forward, the integration of multi‐omics data to reconstruct comprehensive regulatory networks, coupled with rationally designed and well‐powered clinical trials, will be pivotal in ensuring both the safety and therapeutic potential of ncRNA‐based radiosensitisation strategies.

## 3 Conclusion

ncRNAs are increasingly recognised as central players in the development of breast cancer radioresistance, orchestrating complex regulatory networks that govern treatment response. Their dual potential as therapeutic targets and predictive biomarkers opens new avenues for precision radiotherapy.

## Author Contributions

Xiaohui Zhao, Yuting Qiu and Jie Chen conceived the review and edited the manuscript. Danni Wang, Zairui Wang and Shuang Ma contributed to the relevant references collection. Zhuofei Bi, Guoying Liu and Yimin Liu offered guidance and revised the manuscript. All authors read and approved the final manuscript.

## Conflicts of Interest

The authors declare no conflicts of interest.

## Data Availability

Data sharing is not applicable to this article as no data sets were generated or analysed during the current study.
